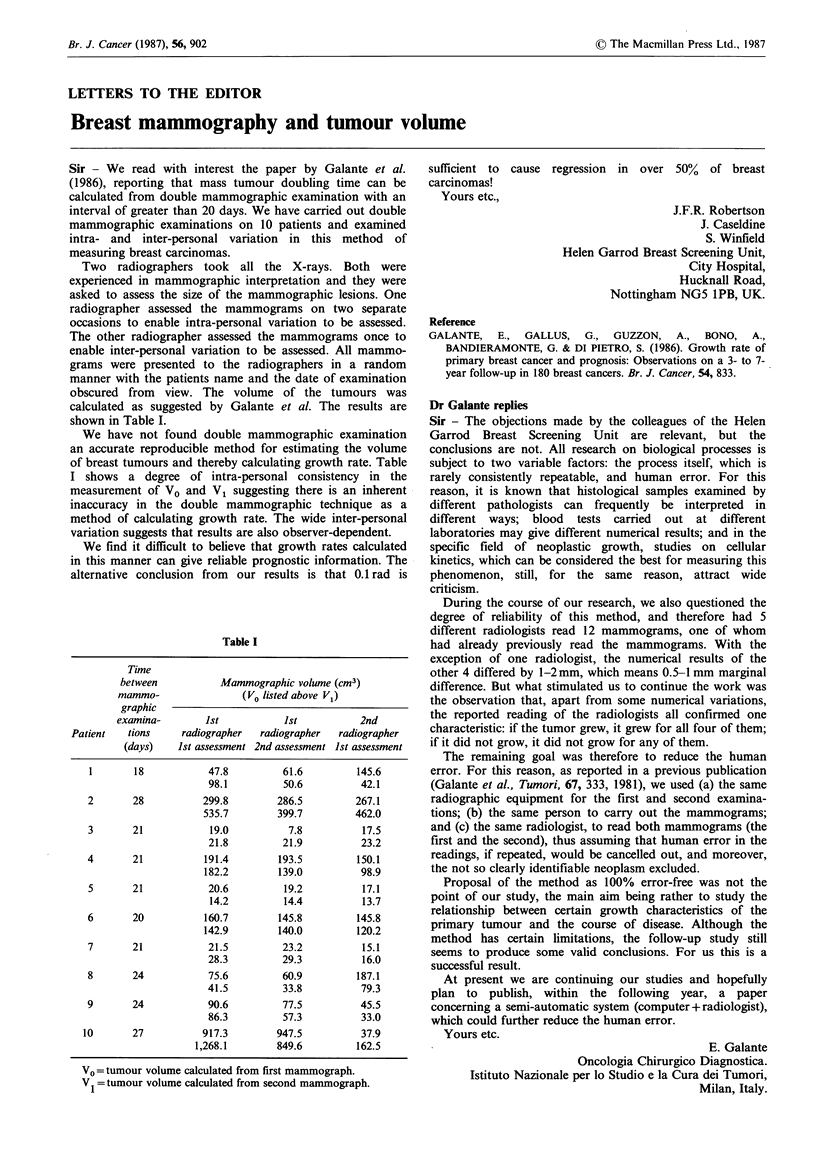# Dr Galante replies

**Published:** 1987-12

**Authors:** E. Galante


Helen Garrod Breast Screening Unit,

### Dr Galante replies

**Sir** – The objections made by the colleagues of the Helen Garrod Breast Screening Unit are relevant, but the conclusions are not. All research on biological processes is subject to two variable factors: the process itself, which is rarely consistently repeatable, and human error. For this reason, it is known that histological samples examined by different pathologists can frequently be interpreted in different ways; blood tests carried out at different laboratories may give different numerical results; and in the specific field of neoplastic growth, studies on cellular kinetics, which can be considered the best for measuring this phenomenon, still, for the same reason, attract wide criticism.

During the course of our research, we also questioned the degree of reliability of this method, and therefore had 5 different radiologists read 12 mammograms, one of whom had already previously read the mammograms. With the exception of one radiologist, the numerical results of the other 4 differed by 1–2 mm, which means 0.5–1 mm marginal difference. But what stimulated us to continue the work was the observation that, apart from some numerical variations, the reported reading of the radiologists all confirmed one characteristic: if the tumor grew, it grew for all four of them; if it did not grow, it did not grow for any of them.

The remaining goal was therefore to reduce the human error. For this reason, as reported in a previous publication (Galante *et al., Tumori,* **67**, 333, 1981), we used (a) the same radiographic equipment for the first and second examinations; (b) the same person to carry out the mammograms; and (c) the same radiologist, to read both mammograms (the first and the second), thus assuming that human error in the readings, if repeated, would be cancelled out, and moreover, the not so clearly identifiable neoplasm excluded.

Proposal of the method as 100% error-free was not the point of our study, the main aim being rather to study the relationship between certain growth characteristics of the primary tumour and the course of disease. Although the method has certain limitations, the follow-up study still seems to produce some valid conclusions. For us this is a successful result.

At present we are continuing our studies and hopefully plan to publish, within the following year, a paper concerning a semi-automatic system (computer + radiologist), which could further reduce the human error.

Yours etc.

E. Galante
Oncologia Chirurgico Diagnostica.
Istituto Nazionale per lo Studio e la Cura dei Tumori,
Milan, Italy.